# Developments in the epidemiology and surgical management of patella fractures in Germany

**DOI:** 10.1186/s12891-023-06162-x

**Published:** 2023-01-31

**Authors:** Yannick Rau, Thomas Huynh, Karl-Heinz Frosch, Carsten Schultz, Arndt-Peter Schulz

**Affiliations:** 1 Faculty of Medicine, Universität Zu Lübeck, Lübeck, Germany; 2 Chair of Technology Management, Christian-Albrechts-Universität, Kiel, Germany; 3 Department of Trauma Surgery, Orthopaedics and Sports Traumatology, BG Klinikum Hamburg, Hamburg, Germany; 4 Department of Trauma and Orthopaedic Surgery, University Medical Center Hamburg-Eppendorf, Hamburg, Germany

**Keywords:** Patella fracture, Plate osteosynthesis, Incidence, Epidemiology, Knee, Technology diffusion

## Abstract

**Background:**

Patella fractures account for approximately 1% of all skeletal injuries. Treatment options are vast and no definitive conclusion on what option is the most beneficial could be made so far. Plate osteosynthesis appears to gain in importance. We aim to give insight into the more recent trends and developments as well as establish the epidemiology of patella fractures in Germany by analysing treatment and epidemiological data from a national database.

**Methods:**

Anonymised data was retrieved form a national database. In the period of 2006 to 2020, all patients with patella fractures as defined in ICD-10 GM as their main diagnosis, who were treated in a German hospital were included. Patients were divided into subgroups based on gender and age. Age groups were created in 10-year intervals from 20 years old up to 80 years old with one group each encompassing all those above the age of 80 years old and below 20 years old and younger. Linear regression was performed were possible to determine statistical significance of possible trends.

**Results:**

A total of 151,435 patellar fractures were reported. 95,221 surgical interventions were performed. Women were about 1.5 times more likely to suffer from patella fracture than men. The relative number of surgical interventions rose from about 50% in 2006 to 75% in 2020. Most surgical interventions are performed in those over the age of 50. The incidence of complex fractures and plate osteosynthesis has significantly increased throughout the analysed period.

**Conclusions:**

We found a clear trend for surgical treatment in Germany with an increase in surgical procedures. We could also show that this ratio is age-related, making it more likely for younger patients in the age groups from 0 to 70 years old to receive surgical treatment for their patella fracture.

## Background

The patella acts as apoint of leverage, augmenting the quadriceps force and thus being of central importance to the extensor mechanism of the knee [1]. Due to its subcutaneous position and high mechanical strain, it is vulnerable to injury. Patellar fractures are relatively rare and occur with an incidence of about 1% of all skeletal injuries [1, 2]. In a large retrospective review of clinical and radiological records of 756 patellar fractures from the north of Denmark treated between 2005 and 2014, the mean age at the time of fracture was 54 years, with an average of 46 years for males and 61 years for females. The sex distribution was 56% females and 44% males. The reported incidence of patellar fractures between 2005 and 2014 was 13.1/100,000/year in this study. AO type 34-C3 was the most common fracture type, representing 25% of all patellar fractures [3]. In most studies examining the incidence in a general population, the incidence is highest for female patients between 60 and 80 years of age, indicating a relationship with osteoporosis [2–6].

Fractures include direct (anterior blunt force), indirect (avulsion due to exceeding stress in tension) or combined trauma mechanisms, which ultimately lead to a partial or complete discontinuance of the extensor mechanism of the knee [1]. Diagnosis is based on the injury mechanism, physical examination, and radiological findings. In the past, the classification of patella fractures resulted from fragment patterns in conventional radiographs, while the diagnostics included traditional two-plane radiographs. In recent years, the use of computerised tomography has shown to improve diagnostic accuracy and influence treatment strategies for patellar fractures [1, 7, 8]. In recent studies, it has been shown that an involvement of the inferior patella pole can be detected in 88% of cases by using an additional preoperative CT scan, whereas these fractures could only be detected in 44% of cases when assessed by conventional X-rays. By using an additional preoperative CT scan, the treatment plan was changed in almost 50% of cases [7]. Therapeutic approaches include conservative and surgical strategies depending on the type and extent of injury [1].

**Fig. 1:**
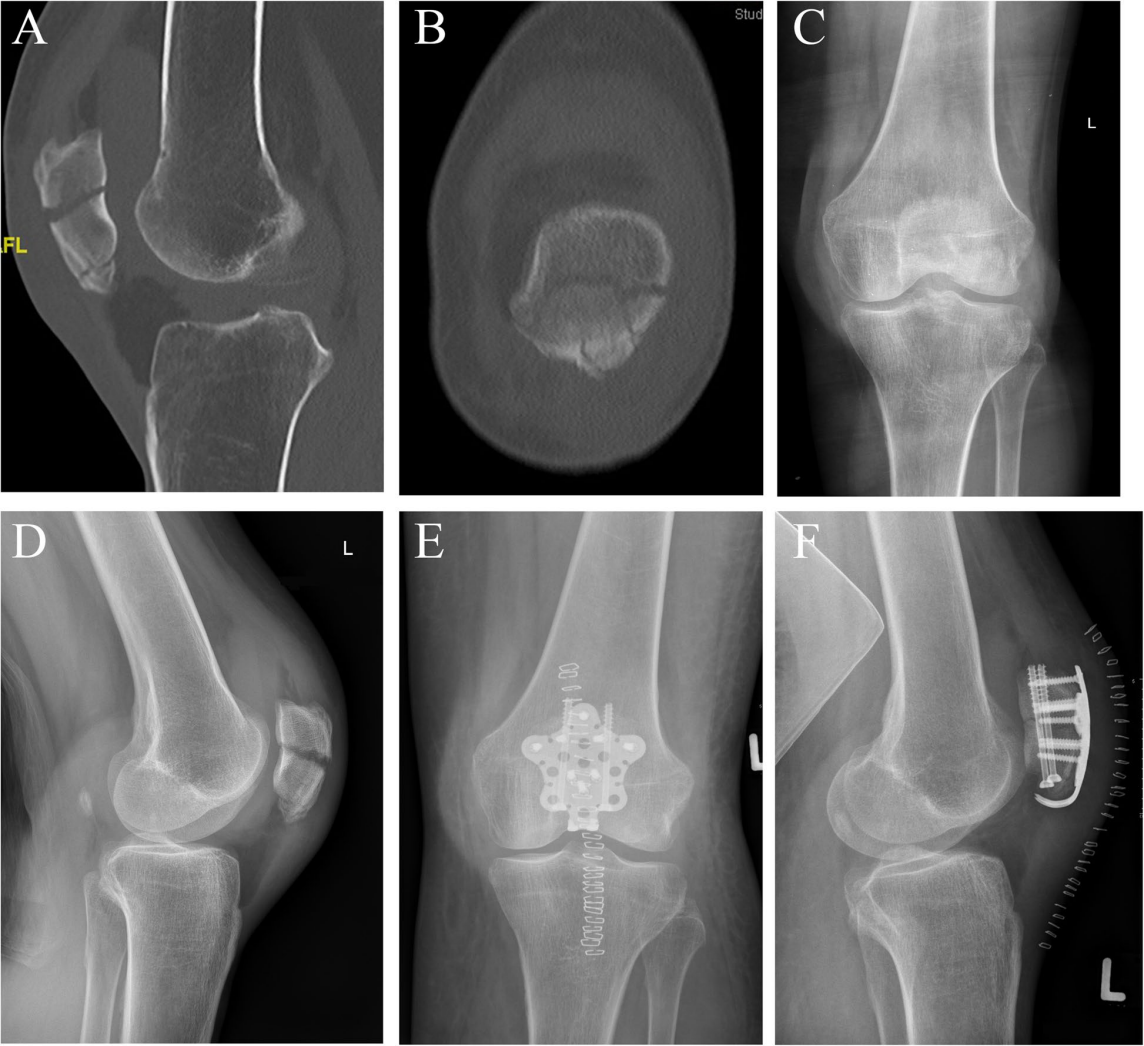
**a**-**f**: patella fracture with involvement of the inferior pole, overlooked in initial radiography but diagnosed in a preoperative CT (**a**—**d**). Postoperative X-ray shows an anatomic reduction with a locked hook plate and compression screws (**e**–**f**)

Regarding the optimal treatment of patella fractures, no general consensus exists [9–11]. Tension band wiring supplemented by Kirschner wires is in longstanding use, with cannulated screws also often being applied. Previous studies showed a significantly higher stability of fixed-angle or locked patella plates compared to tension wiring with K-wires and cannulated lag screws [12, 13]. Plate osteosynthesis has been advocated as a treatment method for this reason [1]. The first reports of this technique using locking plate technology was described in 2011 [14]. There have been increasing numbers of scientific publications regarding the treatment of patellar fractures using plate-osteosynthesis methods over the last decade, often using locked plate technology (Fig. 1a-f) and/or hook extensions [14–20].

Therefore, we asked: 1) are there general trends in the surgical treatments and occurrence of patellar fractures in Germany detectable and 2) do the surgical treatments differ based on patient characteristics, such as age, fracture types and other related factors? With our study, we aimed to establish the epidemiology of patellar fractures to assess whether this novel technology of plate osteosynthesis is a general surgical trend in the treatment of patellar fractures in a large cohort of patients.

An analysis will provide reliable data that can be compared to international developments and can be used to assess economic implications on a nationwide scale. This becomes more and more important as the economic burden on statutory health insurance in Germany is steadily increasing while large scale insights from a clinical perspective are distinctly lacking.

## Methods

### Data collection

Data were retrieved form the German Federal Bureau of Statistics (Statistisches Bundesamt, DESTATIS), Department of the Interior. No administrative permissions to access the data were required and no secondary database was used in this study. The Federal Bureau of Statistics collects anonymized basic demographic data and ICD-10 GM and OPS codes (the German equivalent to ICHI, the International Classification of Procedures in Medicine) for all patients admitted to all German hospitals every year in their database (DRG statistics) and is therefore able to provide detailed information on how many patients were treated as a result of distinct illnesses or injuries as well as to provide details regarding surgical procedures performed [21]. While this includes virtually all inpatient treatments, outpatient treatments in non-hospital facilities are not recorded. Both classification systems are clearly defined, solely descriptive tools, that utilize the performed and validated diagnostic or treatment methods. Both systems are necessary to assess the economic burden of a patient’s treatment and to streamline his diagnoses and procedures when communicating to other health care providers.

Datasets from 2006 to 2020 for all patients being released from hospitals with an S-code main treatment diagnosis corresponding to the WHO group XIX (“injury, poisoning and certain other consequences of external causes”) were provided and a specific injury type was isolated. In particular, ICD-10 GM code S80.2 for patellar fractures was identified.

### Statistical analysis

These numbers were supplied anonymised and subdivided by patient sex and age in 5-year intervals. For this study, patients under the age of 60 were summarised and patients over 60 years were categorised into 10-year intervals. The osteosynthesis methods in the study were extracted from all OPS codes, coding osteosynthesis methods for fracture treatment. All codes involving the treatment of the patella fractures were identified (including: 5–790.0j. 5–790.1j. 5–790.kj. 5–790.nj. 5–790.xj. 5–793.1j. 5–793.2j. 5–793.3j. 5–793.kj. 5–793.xj. 5–794.0j. 5–794.1j. 5–794.2j. 5–794.7j. 5–794.kj. 5–794.xj). These were subdivided into minimally invasive methods, treatment of simple fractures (AO A1 to C1) and treatment of complex fractures (AO C2 and C3). The subdivisions were created by using the procedural codes’ definitions. Details such as the actual AO classification or other fracture classification systems were not sampled.

In addition, we evaluated the data via linear regression analysis and the R based Software Solution Jamovi (Version 2.2.5, The Jamovi Project, Sydney, Australia). In each analysis, time as represented by the year of report or age was analysed as the predictor variable while incidence was chosen as the outcome variable. No additional covariates were used. Linear regression was chosen as a linear approximation of change in incidences was estimated in a first revies of the data. An acceptable alpha error probability was determined at 0.01 as we are conducting research on a large-scale cohort and seek to reduce the probability of declining the null hypothesis because of minimal effects. Model fitness is presented by adjusted R^2^. Furthermore, an F test was performed on each analysis to further determine the significance of the modelled coefficients. 95% Confidence Intervals (CI) are reported if appropriate.

## Results

In the period from 1st of January 2006 to 31st of December 2020, a total of 151,435 patellar fractures (S82.0) were reported to be the main diagnosis of hospitalised patients. This excludes patients suffering from patellar fractures as an accompanying injury of other, more relevant illnesses or injuries.

Total occurrence of patellar fractures and average incidences divided by age and gender are displayed in Table 1.

**Table 1:** Mean incidences for sex and age groups for the whole period

	**Overall**	**Female incidence**	**Male incidence**	** < 21**	**21–30**	**31–40**	**41–50**	**51–60**	**61–70**	**71–80**	** > 80**
Mean	12.222	14.890	9.427	3.375	5.476	5.171	6.216	11.219	19.792	33.042	42.392
95% CI mean lower bound	11.942	14.588	8.831	3.043	4.884	4.817	5.648	10.575	19.045	32.256	41.383
95% CI mean upper bound	12.502	15.192	10.024	3.708	6.069	5.526	6.785	11.864	20.539	33.828	43.401
Median	12.276	14.979	9.611	3.309	5.362	5.313	6.196	11.407	20.254	33.254	42.737

**Fig. 2 a:**
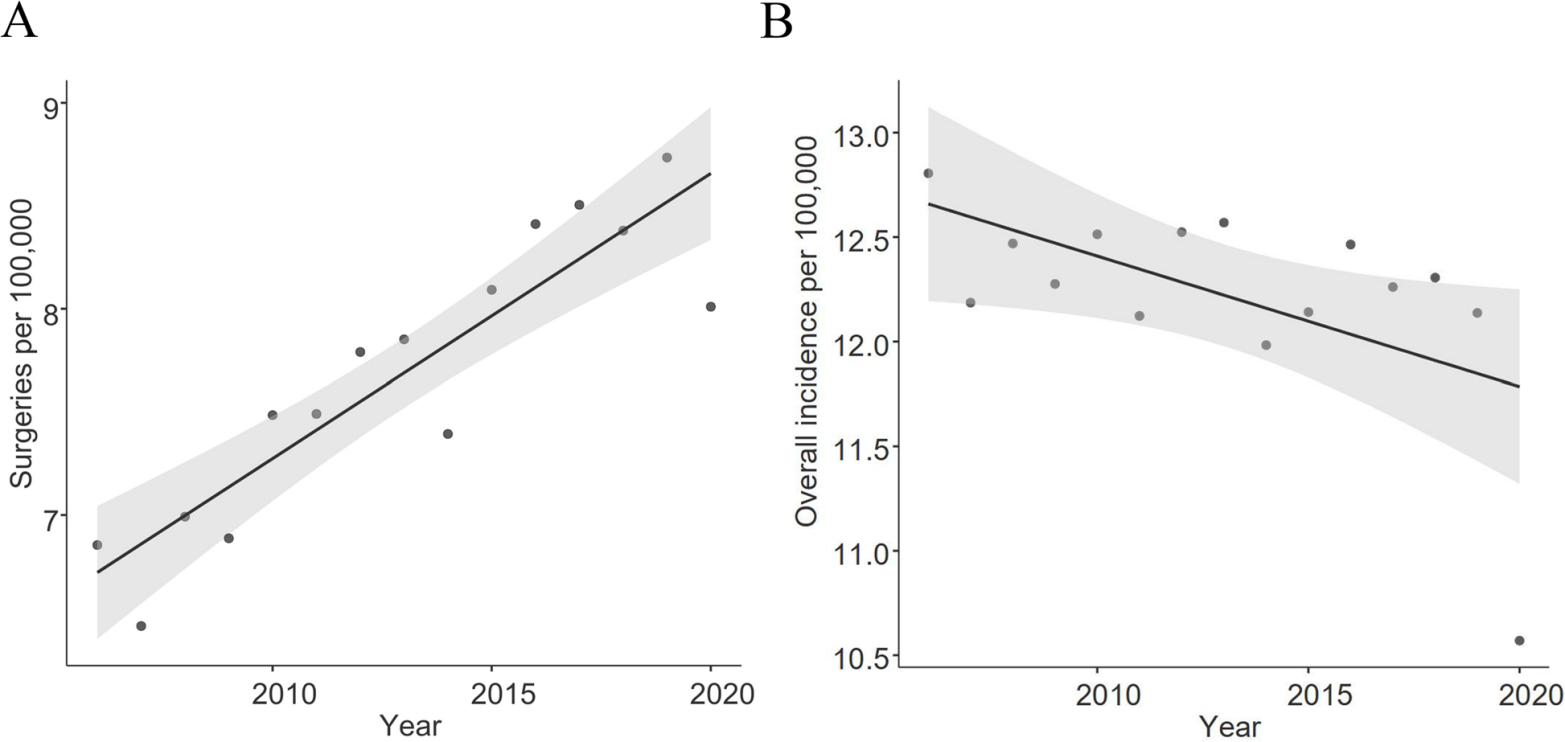
and **b**: Documented annual surgical treatments and occurrence of patellar fractures over 15 years in Germany

A total of 95,221 surgical interventions were performed on the included patients, with an increase in later years, while overall diagnoses remained steady with a slight decrease in occurrence. In 2006, 53.2% of all cases of patellar fractures received a surgical intervention (12.81/100,000 patella fracture diagnoses and 6.81/100,000 surgical procedures), whilst this increased to a share of 75.8% in 2020 (10.57/100,000 diagnoses and 8.01/100,000 surgeries). Confidence intervals are not reported as no means were calculated. An annualised illustration of the development is shown in Fig. 2a and b. The population size adjusted increase in surgical approaches was determined to significantly correlate with time at F(1,13) = 52.12; *p* < 0.001 with an adjusted R^2^ of 0.803 and a correlation coefficient of 0.138 (95%-CI: 0.099, 0.178) patients per 100,000.

**Fig. 3:**
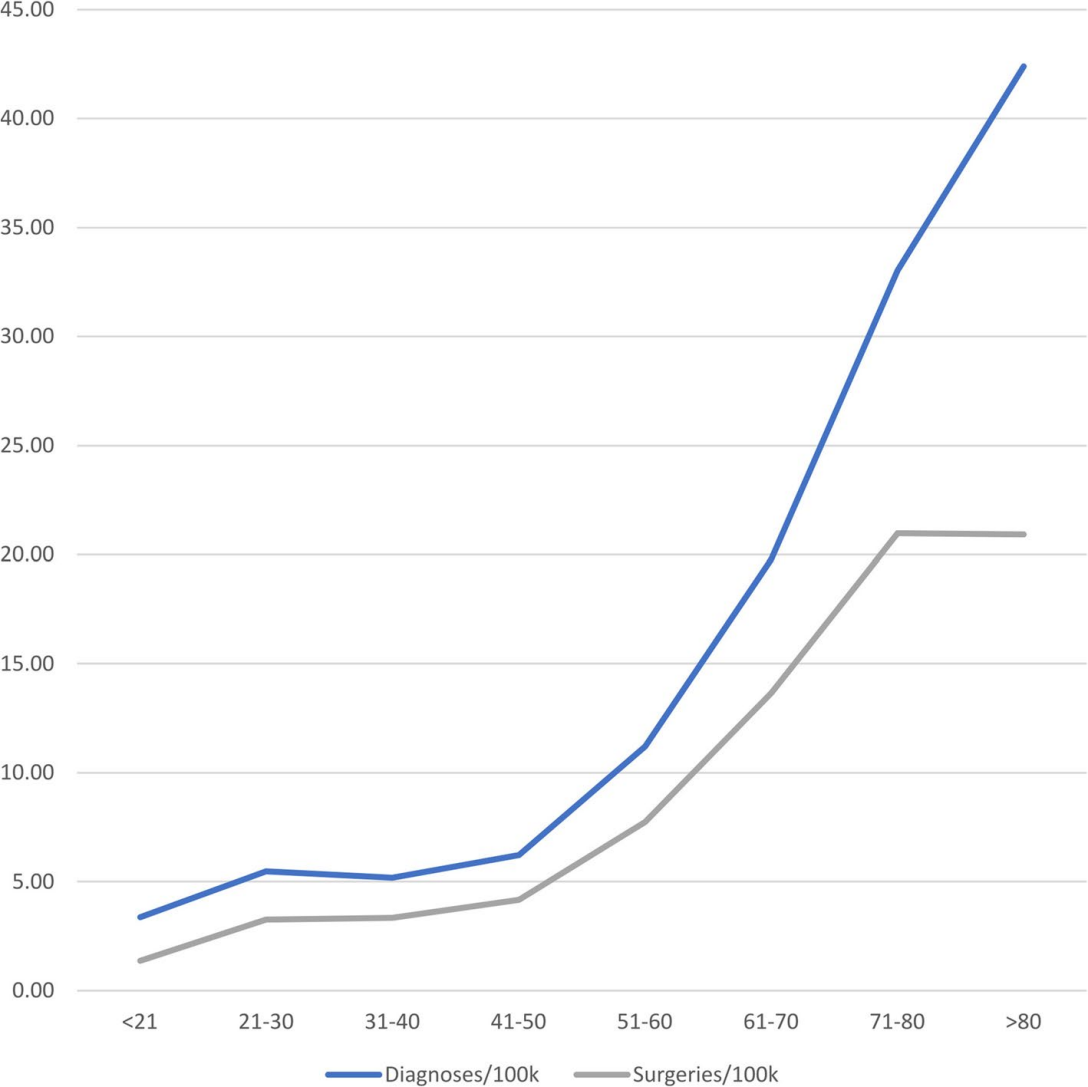
Age distribution of patellar fractures and associated surgery per 100,000 capita per year

Figure 3 shows the overall distribution of performed surgeries in comparison to reported diagnoses over the complete period divided by age groups. While diagnostic incidence is increasing until death, surgical interventions continue to stall at about 70 years and older. The share of surgical interventions increases into the 51–60 age group with 69.1% and 7.75/100,000 (95%-CI: 7.47/100,000, 8.04/100,000) surgeries performed. After that, the relative number of surgeries decreases until it reaches 49.3% in those over the age of 80 with 20.91/100,000 (95%-CI: 20.02/100,000, 21.81/100,000) surgeries performed. The incidence of surgical approaches correlates significantly with age-group at F(1,5) = 52.12; *p* = 0.004 with an adjusted R^2^ of 0.799 and a correlation coefficient of 3.002 (95%-CI: 1.452, 4.553) patients per 100,000 per group.

**Fig. 4 a:**
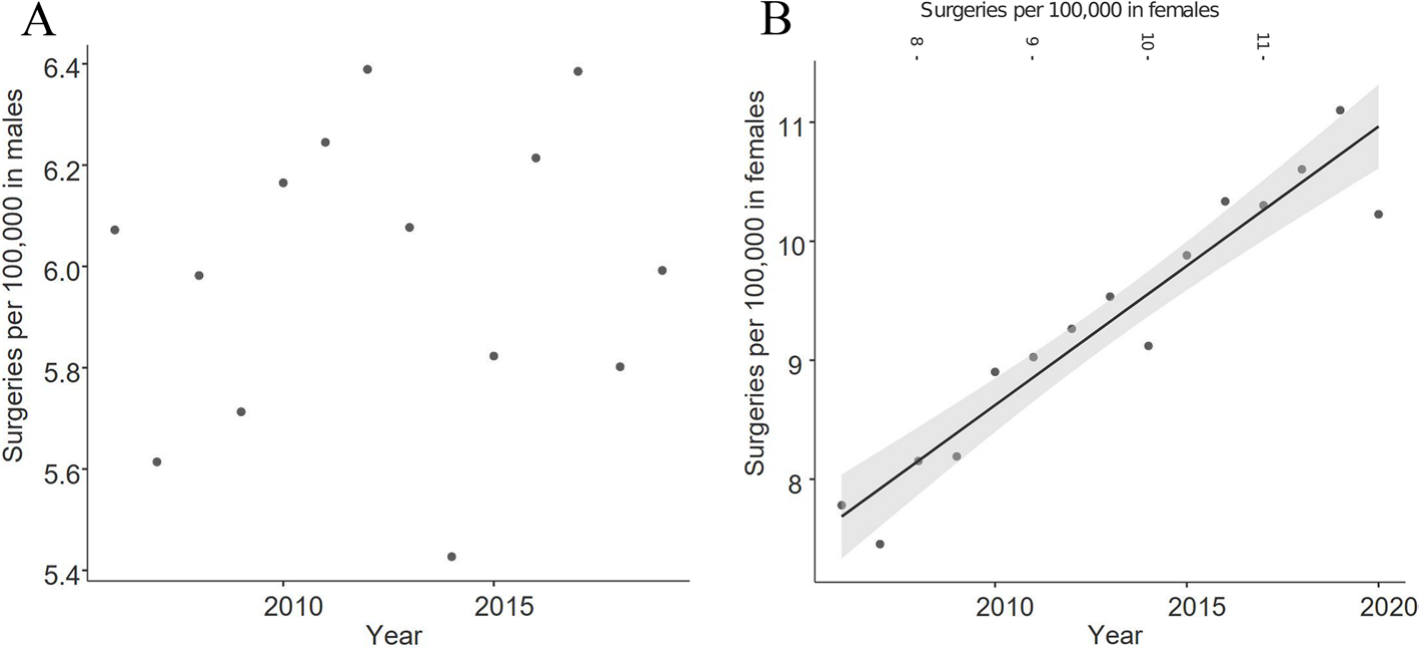
and **b**: Surgery incidence per 100,000 capita per year among male and female patients

A population size adjusted analysis showed that an increase of surgery performed on female patients is predominantly responsible for the increase in overall surgical interventions (Fig. 4a and b). No significant correlation between year and incidence among male patients could be determined. However, the incidence of surgical approaches of female patients correlates significantly with the year of report at F(1,13) = 139.022; *p* < 0.001 with an adjusted R^2^ of 0.908 and a correlation coefficient of 0.234 (95%-CI: 0.191, 0.277) patients per 100,000 per year of report.

**Fig. 5:**
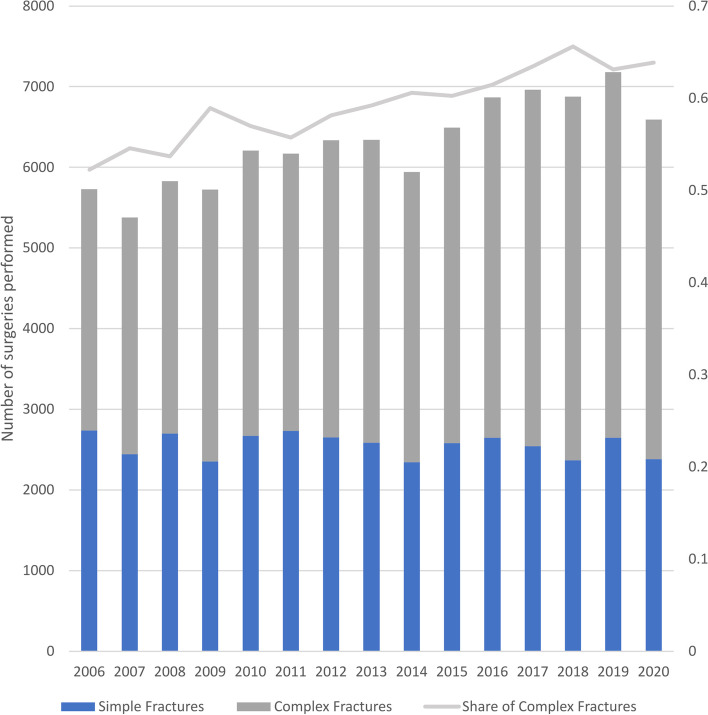
Increase in complex fracture types over time; the percentage of complex fractures is overlaid with values on the right y-axis

Further analysis of the injury types showed that not only the overall amount of surgical interventions performed but also the relative share in complex fracture types is gradually increasing. While only 52.0% of all injuries were classified as complex in 2006, this share steadily increased to 64.0% in 2020 (Fig. 5). This development is mainly caused by a relative increase in the report of complex injuries in those over the age of 60 years-old. The effect shown is equally prevalent in both genders. The overall relative increase in complex fracture treatment reports was significant at F(1,13) = 102.502; *p* < 0.001 with an adjusted R^2^ of 0.879 and a correlation coefficient of 0.8% (95%-CI: 0.7%, 1.0%).

**Fig. 6:**
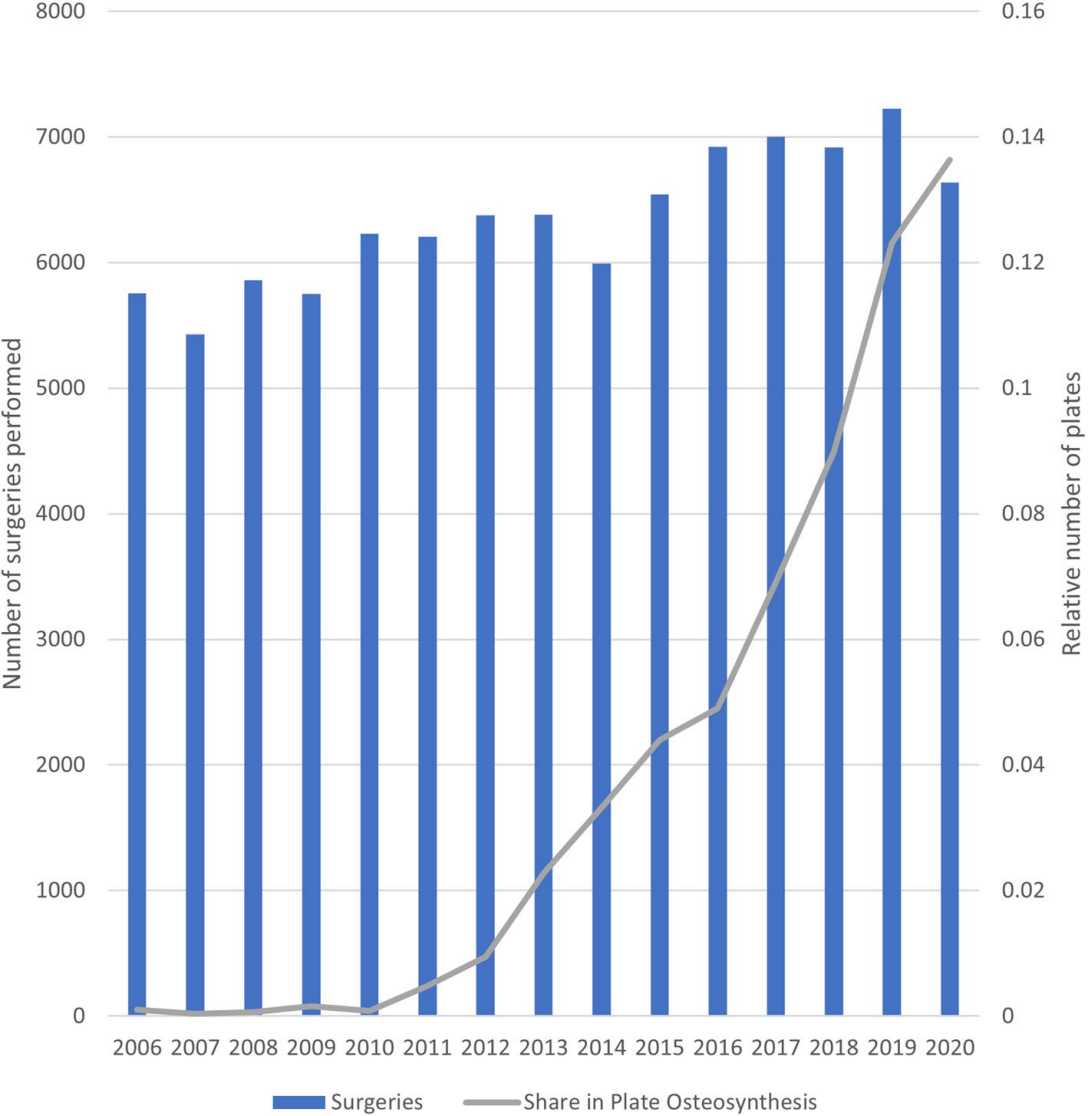
The share of plate osteosynthesis in all patellar fracture types over time

The most common surgical interventions in the early study period involved tension band wiring and screws, while plate osteosynthesis was not being routinely performed. In 2006 only 6 plate osteosyntheses of the patella were reported. From 2010 to 2020, that number increased steadily and rose to 905. Figure 6 illustrates the rising importance of plate dependent systems. The increase in plate osteosyntheses was again determined to be significantly correlated to the year of report at F(1,13) = 63.668; *p* < 0.001 with an adjusted R^2^ of 0.817 and a correlation coefficient of 65.225 (95%-CI: 47.565, 82.885) patients.

Of all 5,036 plate osteosyntheses reported, only 986 (19.6%) were used on simple fractures, whilst the remaining were employed on complex fracture types. The share of plate osteosynthesis in complex fracture treatments rose from 12 (0.4%) in 2006 to 897 (20.4%) in 2020, while its share of simple fracture treatment rose from 4 (0.2%) in 2006 to 197 (8.3%) in 2020. Both absolute increases can be determined as significantly correlated to the year of report over the entire period.

The OPS coding system does only allow to differentiate between fixed-angle and other plates not between individual types of plates themselves. Taking this into account, the majority of plates were reported to be locked or fixed angle plates. The increase in plates used was directly linked to the increase in usage of locked plates in particular as its usage increased from 3 (18.8%) of 16 overall plates in 2006 to 876 (80.1%) of 1094 overall plates in 2020.

**Fig. 7:**
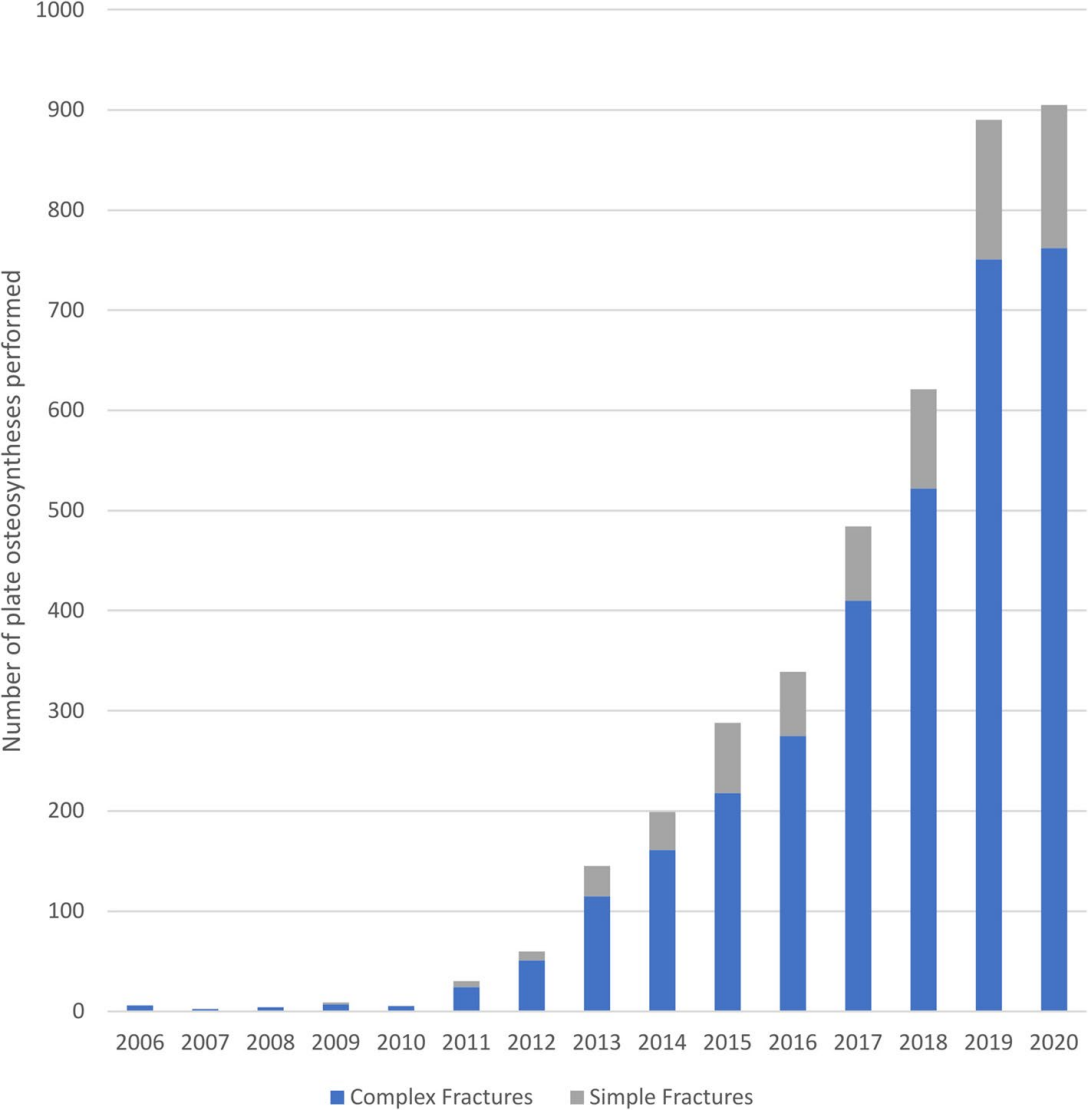
Plate Osteosynthesis in complex and simple fractures

The illustration in Fig. 7 shows the development in the share of plate osteosynthesis in simple fractures. The development shows that the relative number of plates in each type of fracture is remaining steady at about 20% for simple fractures from 2012 to 2020. The lack of usage of plate osteosynthesis in patellar fractures before that time makes the earlier data negligible in this regard.

## Discussion

This study revealed a number of previously unknown facts regarding the epidemiology and development of patellar fractures and their treatment in the last two decades in Germany.

Regarding general epidemiological evaluation, an incidence of 9.43/100,000/year for males and 14.89/100,000/year for females over the complete study period 2006–2020 are similar to previous findings (Table 1). The difference between males and females may be mainly caused by a higher incidence of osteoporosis in the older female population, significantly increasing the risk of fall related fractures as previously reported by other authors [22, 23].

The observed significant increase in fracture incidence among females over time and the lack thereof in the male population as shown in Fig. 4a and b may also be caused by an increased incidence of risk factors associated with fractures among the female population. The main risk factor remaining osteoporosis which’s prevalence has risen together with the aging population while an increase in incidence that would translate to our observed increase in fractures cannot yet be properly backed up by available data [24].

An in-depth analysis of this and other potential risk factors may result in new opportunities to implement preventative strategies. The gender specific trend of increased fracture risk may be of particular importance for future interventions.

In a similar study from Denmark covering the years 2005–2015, the reported overall incidences for males and females were 11.4/100,000/year and 14.7/100,000/year, respectively [3].

In a recent study observing all treatment episodes from 2009 to 2019 from all German medical institutions, the overall incidence of patella fractures was reported to be 14.1/100,000 inhabitants [25]. In this study, the incidence was calculated as 48.1/100,000 for females between 70–79 years (male incidence was 14.8 in that age group), peaking at 54.1/100,000 for females in the age group between 80 and 89 years (the incidence in males was 24.5/100,000).

Regarding this age distribution, Begner et al. has previously described a changing age pattern of patellar fractures in Malmö in 1986, with an age peak of 35–44 years for males and 55–64 years for females in the 1950s, developing to age peaks of 55–64 years in males and older than 74 years in females in the 1980s [5]. This continues with a clear age peak over 80 years of age in the current study for Germany.

The annual ratio of conservative versus surgical treatment has never been reported until now in a study using epidemiological data. The Swedish Fracture Registry recently reported about 3194 patella fractures occurring over a 5-year period [26]. In this cohort, 2138 (66.9%) of cases were treated conservatively. In a study of the Danish fracture registry covering all fractures of the knee observing a 20-year period from 1997, a surgical treatment rate of 26.3% was determined for patella fractures.

We found a clear trend for surgical treatment in Germany with an increase in surgical procedures, rising from a surgery rate of 53.16% in 2006 to 75.78% in 2020. We could also show that this ratio is age-related, making it more likely for younger patients below the age of 71 to receive surgical treatment for their patella fracture. Furthermore, our data analysis showed that an increase in surgery performed on female patients is predominantly related to the increase in overall surgical interventions over the study period. The reason for the higher share of surgical treatment in comparison to Sweden or Denmark could be, next to a higher affinity to surgical treatment in Germany, the non-detection of very simple fractures that might not receive a diagnosis in an emergency department or surgical clinic in Germany. Cases that are solely handled by an ambulatory general practitioner are also not registered in the system and could therefore be underreported in our study.

When searching for reasons for this changing pattern, we evaluated different injury types. We could show that the relative share in complex fracture types is gradually increasing by time. While only approximately 52% of all treatments were classified as complex fracture types in 2006, this share steadily increased to about 64% in 2020 (Fig. 4). This development is mainly caused by a relative increased report of complex injuries in those over the age of 60 years old. The data sampled by DESTATIS only allows authors to distinguish between surgeries performed for simple versus complex patellar fractures. No further classifications or subtypes are sampled. Again, comparing this data to the Swedish Registry Data, approximately 64% complex fractures were found in our study compared to 72% for Sweden [26]. This demonstrates that the underreporting of simple fractures does not appear to occur in our study. This makes an influence of the increasing use of computed tomography, often including 3D reconstruction, very likely. In previous studies, the use of computed tomography improved the interrater reliability of the AO/OTA classification in patellar fractures with a further improvement when using three-dimensional techniques [27]. Another study comparing radiography with computed tomography in the diagnosis of patellar fractures showed an upgrade of fracture complexity in almost 50% of cases [7]. In a recent study evaluating the fracture types judged by CT scan in a consecutive cohort of 200 patients from Korea, AO C-type fractures were found in 2/3 of cases [28]. Overall, this makes increasing mean age as well as improved diagnostic accuracy the highly likely combined factors for complex patellar fractures.

Another potential explanation for an increase in complexity of fractures may be due to a change in the frequency of injury causes. High energy trauma like those suffered when involved in a road traffic accident is such a potential cause as traffic accidents have previously been reported as one of the leading causes of traumatic patella fractures and are more commonly high energy in nature than simple falls, the most common cause for patella fractures [6, 26, 29]. To evaluate a possible connection, we used the data on traffic accidents collected by the federal department of the interior on the frequency of road traffic accidents in Germany. We discovered that while in 2006, 327,984 road traffic accidents involving personal injuries were reported to the police, this number actually decreased up until 2020 with 264,499 accidents reported. Even when considering the Covid-19 pandemic and its associated reduction in road traffic and taking a look at the reported data of 2019, the number of accidents still fell short with 300,143 [30, 31]. This decrease in accident frequency in addition to our observed increase in complex fracture incidence make a potential direct correlation of the frequencies unlikely. It has to be noted though that newer modes of transportation like pedelecs or power-driven scooters may play a role in injury frequency and severity involved in present and future traffic accidents, so that even a reduced frequency but an associated shift in injury patterns may influence or have influenced the observed development. For example, while overall bicycle accidents have remained steadily frequent, pedelec related accidents have been reported to have increased between 2012 to 2020 with a factor of over 40 [32]. However, one monocentric study from an emergency department in the Netherlands including 78 patients for example could not find significant differences between pedelec riders and conventional cyclists with regards to overall mechanisms of injury and injury severity [33]. More research on the types and biomechanics of injury, especially of the knee joint, is needed to make a definitive and sufficiently supported statement on the issue of the observed increase of complex fractures of the patella.

The initiation of this study was influenced by an observation of a possible increase in plate osteosynthesis treatment in recent decades. Indeed, our results could confirm the increased use of this technique over the last decade. This increase appears to follow an increase in more complex fracture types, especially in the osteoporosis-prone group of females in the age group over 70 years. The evaluation showed that plate osteosynthesis procedures in the vast majority are employed in those complex fracture types whilst simpler fractures tend to be managed conservatively or by simpler surgical techniques such as tension band wiring or compression screw osteosynthesis. The increasing use of locked patellar plates appears to be driven by the clinical needs of these fracture types.

The minimization of involved risks and potential revisions may be the most influential factor within the development of treatment options. It has been previously reported that plating provides an increased biomechanical stability and better clinical outcome parameters than the more commonly used tension band wiring techniques. Therefore, the more common use of plating techniques and the fixed angle or locked plate in particular should result in a reduction of revisions and also improve patient safety and long-term outcome [14, 15, 34–36].

An apparent tendency to produce fracture displacement under load seems to be a major drawback of tension wiring when compared to fixed angle-plates for example [35, 36].

Additionally, wiring and tension banded osteosynthesis are associated with a high incidence of and need for secondary implant removal surgery ranging from about 27 to 40% due to pain, irritations of the skin or infections [37, 38]. This rate may be drastically reduced by implanting plates instead of Kirschner wires or similar options.

As a clear limitation of this study, it has to be stated that no outcome data are available, making it impossible to state whether the application of plate osteosynthesis, or indeed surgical treatment at all, is beneficial in complex fractures of the patella. This requires controlled randomised trials comparing these treatment forms or long-term registries with follow-up periods that cover possible long-term developments such as the necessity for total knee arthroplasty for posttraumatic knee osteoarthritis.

Additionally, both underlying coding systems are the basis of all economic compensations between the statutory health insurance and public health care providers in Germany. Compensation may depend on clinical severity of injury and complexity of treatment. The assessment via code therefore not only has clinical but also economic implications. It is heavily reliant on the individual physician's or surgeon’s assessment. A potential cause for an increase in the diagnosis of complex patella fractures may also be the financial imbursement difference when compared to simple fractures. Dependent on the complexity of surgery and diagnosis, reimbursement may vary drastically.

This may result in an incentive to further investigate the complexity of fractures by including otherwise not implemented diagnostic modalities like computed tomography. In some cases, this may even lead to a falsification of codes associated with the underlying diagnosis to achieve higher reimbursement rates. This phenomenon is also known as “upcoding” and was reported as one of the main problems with reimbursement in the German health economy in the early 2000s [39, 40].

This issue has been thoroughly addressed by the proper authorities in the past and is by no means a tolerated practice as it undermines the reimbursement system and objectivity of health care data as a whole.

But, while knowingly altering the assessment for monetary gain would be considered illegal, a possible bias towards more complex treatment modalities should not be discarded outright.

## Conclusions

We were able to provide incidence data on patellar fractures on a nationwide scale. We discovered an increase in surgical procedures while overall diagnoses remained on a steady sideward trend. The increase in surgeries is mainly affecting female patients as their increased share in surgical treatments is not explainable by their sideward trend in overall diagnoses. Additionally, we discovered that the increase in surgeries is aligning with the broad scale implementation of plate osteosynthesis. We could also show that this ratio is age-related, making it increasingly more likely for younger patients below the age of 71 to receive surgical treatment for their patella fracture.

Also, there seems to be a steady increase in the share of complex patellar fractures. These fractures require additional treatment efforts and more complex surgical interventions. This should lead to further investigation for possible preventative approaches as no apparent explanation for this increase could be found.

## Data Availability

The datasets generated and/or analysed during the current study are available from the corresponding author upon reasonable request. Raw data concerning the German hospital statistics are also available upon request directly from the federal bureau of statistics or partly from its health-reports website gbe-bund.de.

## Abbreviations

AO/OTA: Arbeitsgemeinschaft für Osteosynthesefragen/Orthopaedic Trauma Association
CT: Computed tomography
WHO: World health organisation
CI: Confidence interval
ICD-10 GM: International classification of diseases German modified
OPS: Operationen und prozedurenschlüssel
ICHI: International classification of health interventions
DRG: Diagnosis related groups
